# A Novel Mutation in the NBD Domain of *NLRC4* Causes Mild Autoinflammation With Recurrent Urticaria

**DOI:** 10.3389/fimmu.2021.674808

**Published:** 2021-06-23

**Authors:** Li Wang, Wen Wen, Mengyue Deng, Yue Li, Gan Sun, Xiaodong Zhao, Xuemei Tang, Huawei Mao

**Affiliations:** ^1^ Department of Pediatric Research Institute, Chongqing Key Laboratory of Child Infection and Immunity, Ministry of Education Key Laboratory of Child Development and Disorders, National Clinical Research Center for Child Health and Disorders (Chongqing), China International Science and Technology Cooperation Base of Child Development and Critical Disorders, Children’s Hospital of Chongqing Medical University, Chongqing, China; ^2^ Department of Rheumatology and Immunology, Children’s Hospital of Chongqing Medical University, Chongqing, China; ^3^ Department of Immunology, Ministry of Education Key laboratory of Major Diseases in Children, Beijing Children’s Hospital, Capital Medical University, National Center for Children’s Health, Beijing, China

**Keywords:** NLRC4, novel mutation, autoinflammatory disease, mild phenotype, urticaria

## Abstract

**Background:**

NOD-like receptor family CARD-containing 4 protein (NLRC4) is a cytosolic protein that forms an inflammasome in response to flagellin and type 3 secretion system (T3SS) proteins from invading Gram-negative bacteria. *NLRC4* mutations have been recently identified in early-onset severe autoinflammatory disorders. In this study, we reported a novel mutation in *NLRC4* in two Chinese patients, who manifested with recurrent urticaria and arthralgia.

**Methods:**

We summarized the clinical data of the two patients. Gene mutations were identified by whole-exome sequencing (WES). Swiss-PdbViewer was used to predict the pathogenicity of the identified mutations. Cytokine levels and caspase-1 activation were detected in the patient PBMCs with lipopolysaccharide (LPS) stimulation. All previously published cases with *NLRC4* mutations were reviewed.

**Results:**

We identified a missense heterozygous mutation (c.514G>A, p.Gly172Ser), which was located in the highly conserved residue of nucleotide-binding domain (NBD) of NLRC4. The mutation did not alter the expression of NLRC4 protein, but induced considerably much higher production of IL-1β and IL-6 in patient PBMCs than in healthy controls after LPS stimulation. Four NLRC4 inflammasomopathy phenotypes have been described, with severe inflammatory diseases including macrophage activation syndrome, enterocolitis and NOMID in patients with mutations in the NBD and HD1 domains, whereas a mild clinical phenotype was associated with two mutations in the WHD domain of NLRC4.

**Conclusion:**

We identified a novel mutation in the NBD domain, and the patients just presented with a mild inflammatory phenotype. Thus, our findings reinforce the diversity of *NLRC4* mutations and expand the clinical spectrum of associated diseases.

## Introduction

Monogenic autoinflammatory diseases (AIDs) are a heterogeneous group of diseases, characterized by hyperactivation of the innate immune system without apparent involvement of either autoantibodies or autoreactive T cells (1, 2). Gain-of-function mutations in the inflammasome forming proteins, such as NOD-like receptors (NLRs), are a major cause of monogenic autoinflammatory disorders (3–5). Inflammasomes are innate immune sensors that respond to pathogen- and damage-associated signals with caspase-1 activation, interleukin (IL)-1β and IL-18 secretion, and macrophage pyroptosis. Overactivation of inflammasomes leads to systemic autoinflammatory diseases (6, 7).

Gain-of-function mutations in the *NLRP3* gene are well known to cause cryopyrin-associated periodic syndromes(CAPSs) including familial cold-induced autoinflammatory syndrome (FCAS, OMIM #120100), Muckle Wells syndrome (MWS, OMIM #191900) and neonatal-onset multisystem inflammatory disease (NOMID, OMIM #607115) (3, 8). The IL-1β inhibitors, such as anakinra, canakinumab, and the IL-1 receptor type I fusion protein rilonacept, induce significant clinical response in CAPS, indicating that signal transduction through the IL-1 receptor is crucial for the pathogenesis of CAPS (9, 10).

Recently, gain-of-function mutations in *NLRC4* were reported to cause spontaneous inflammasome activation in the absence of infections (11, 12). Most of the mutations are located in the nucleotide-binding domain (NBD) and helicase domain 1 (HD1) of NLRC4, and induce severe autoinflammation with high levels of IL-18 in the serum of the patients. These mutations lead to life-threatening macrophage activation syndrome (MAS) and severe enterocolitis, also called autoinflammation with infantile enterocolitis (AIFEC; OMIM #616050) (13). In addition, two mutations in wing helix domain (WHD), p.H443P and p.S445P were reported to cause a clinical phenotype similar to FCAS1, mainly with recurrent rash and joint symptoms (FCAS4; OMIM #616115) (14, 15). There may be a genotype/phenotype correlation between the amino acid position of the NLRC4 mutation and the phenotypes described. Mutations within the NBD and HD1 domains are associated with severe phenotypes including MAS and AIFEC, whereas those within the WHD domain were found in patients with mild inflammatory symptoms such as FCAS4.

In this study, we identified a novel mutation in the NBD domain of *NLRC4* in two patients, who presented just with recurrent urticaria and arthralgia. This is the first report of FCAS4 associated with a mutation in the NBD domain of *NLRC4*. We further demonstrated the diversity in the *NLRC4* variation and clinical phenotype, thus expanding the understanding of disease spectrum. In addition, we conducted a literature review of NLRC4 cases published up until June 2020, and described the genetic, clinical manifestations and the treatment of carriers of these mutations.

## Methods

### Patient and Study Approval

There were two patients enrolled in this study. The proband was a 4-year-old boy and the other patient was his mother, both of them manifested with recurrent urticaria and arthralgia. Clinical data, family history and blood samples were collected when the proband first visited us in May 2019. Informed consent was obtained from all of the participants. This study was conducted in accordance with the tenets of the Declaration of Helsinki and was approved by the ethics committee of the Children’s Hospital of Chongqing Medical University.

### Genetic Analysis

Peripheral blood was collected from members of this family after written informed consent obtained. Whole blood samples were sent to MyGenostics (Beijing, China) and subjected to whole-exome sequencing (WES). The pathogenicity of the mutations was assessed following the American College of Medical Genetics and Genomics guidelines (ACMG). Mutations in NLRC4 were confirmed by using Sanger sequencing. Briefly, genomic DNA was extracted from peripheral blood mononuclear cells (PBMCs) using the QIAamp DNA Mini Kit (Qiagen, Shanghai, China). Polymerase chain reaction (PCR) was performed to amplify the genomic NLRC4 gene. The products of the PCR reactions were sent for Sanger sequencing.

### Analysis of the NLRC4 Structure

The crystal structure of NLRC4 (4KXF from the Protein Data Bank) was used as the template, which was determined by X-ray diffraction at a resolution of 3.20 Å (16, 17). Although the 4KXF is the crystal structure of mouse NLRC4, it does reveal the auto-inhibition mechanism. And previous studies also used it as the template to study the effect of NLRC4 mutation (11, 12, 18). Herein the structural impact of mutant Gly172Ser was analyzed by Swiss-PdbViewer. Residue 172 and certain nearby residues within 3 Å are illustrated. For clear demonstration of the interresidue relationship, some residues were highlighted in the indicated colors with the computed hydrogen bonds being labeled.

### PBMC and Plasma Isolation

Peripheral blood was collected in vacutainers containing sodium heparin. Centrifugation was performed at 2500 rpm for 5 minutes at room temperature to separate the plasma from the peripheral blood cells. The cells were diluted in PBS and added to the top of Ficoll-Paque PREMIUM (GE Healthcare, Sweden) and centrifuged at 800 g for 20 minutes at room temperature to separate the PBMCs fraction.

### Cell Stimulation

PBMCs were cultured in RPMI 1640 (Gibco, USA) at a density of 0.8× 106 cells/ml containing 10% FBS (Gibco, USA), and penicillin-streptomycin (100 U/mL each; Sigma-Aldrich, USA) was added. Lipopolysaccharide (Sigma Aldrich, USA) was added to cell culture at a final concentration of 100 ng/ml for stimulation. After 24 hours of culture, the cells were collected for the subsequent experiments.

### Cytokine Quantification From the Cell-Culture Supernatants

After cell stimulation, the plate was centrifuged to pellet the cells. The presence of cytokines in the supernatants was determined by using Multi-Analyte Flow Assay Kit (Human Inflammation Panel(13-plex), Biolegend, San Diego, CA) according to the manufacturer’s guidelines.

### Caspase-1 Luminescence Assay

Caspase-Glo^®^ 1 inflammasome assay kit (Promega, USA) was used to measure the caspase-1 activation in the PBMCs culture supernatant. Caspase-1 and caspase-1 inhibitor reagents were prepared and added to the supernatant directly as described by the manufacturer. Fifty microliters of supernatant was transferred to a white 96-well plate. Caspase-Glo^®^ 1 Reagent or Caspase-Glo^®^ 1 YVAD-CHO Reagent was added to the wells (50 µl/well) and gently mixed on a plate shaker at 300 rpm for 30 s. The mixture was then incubated for 60 minutes at room temperature before measuring the luminescence on a Berthold Technologies luminometer.

### Western Blot Analysis

Cells were immediately washed with PBS and lysed in RIPA buffer with complete protease inhibitor (Sigma-Aldrich) immediately. Protein lysates in protein loading buffer were separated in 10% SDS-PAGE gels, transferred to PVDF membranes (Millipore, Germany), blocked with 5% milk, and then probed overnight at 4°C with primary antibodies, including anti-NLRC4 (ab115537; Abcam, UK) and anti-GAPDH (Bioss antibodies, Woburn, MA). After incubation with an HRP-conjugated secondary antibody (Bioss, USA), the blots were washed with TBST and imaged using an enhanced chemiluminescence system (Thermo Scientific, USA).

### Methodology for the Literature Review of NLRC4 Mutations

A literature review of NLRC4 mutations was performed in PubMed using the terms “NLRC4” and ‘‘mutation’’. All types of publications (articles, reviews, editorials, letters, and correspondence), written in English, and published online between January 2014 and June 2020 were included. Only pathogenic and likely pathogenic mutations were considered.

### Statistical Analysis

All statistical analyses were conducted with GraphPad Prism 8 software (GraphPad Software, Inc., San Diego, CA). Data were analyzed using an unpaired two-tailed Student t test. P-values < 0.05 were considered to indicate a significant difference.

## Results

### Clinical Phenotype of the Patients

**Figure 1:**
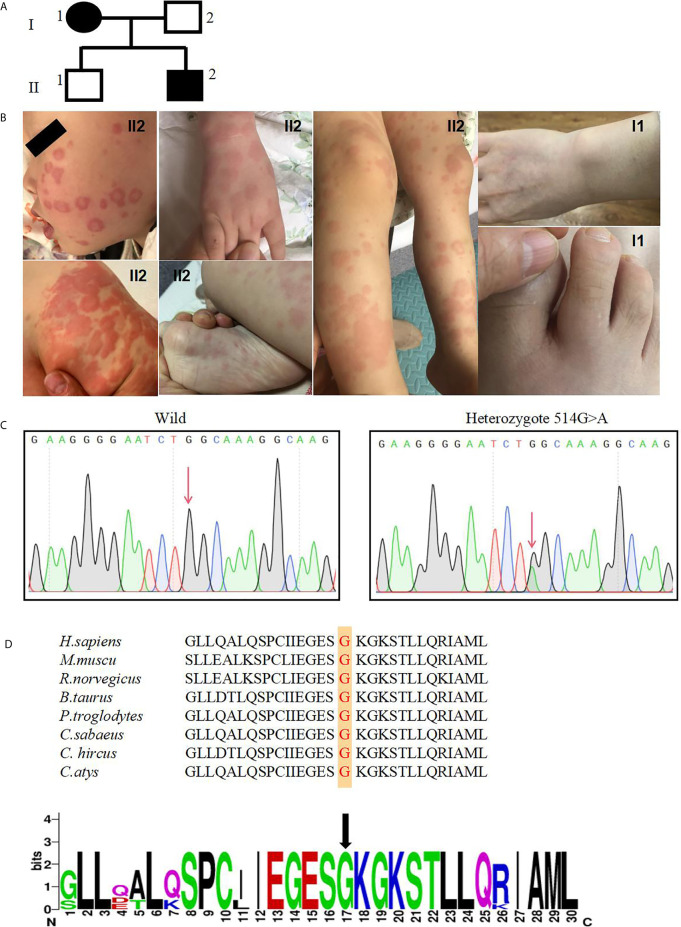
A novel NLRC4 mutation was revealed in the patients. **(A)** The family pedigree. Squares, Male subjects; circles, female subjects. **(B)** Urticarial skin rash in patient II2, photographed at age 6 months and 4 years old: the face, buttock, extremities and soles are affected. Swelling of the first interphalangeal joint of the right toe and the right wrist of patient I1. **(C)** Confirmation of a mutation in NLRC4 by Sanger sequencing. **(D)** The amino acid sequence at position 172 (red) of NLRC4 in eight species and indicated by the black arrow.

The proband patient (**Figures 1A** **, II2**) in our study was a boy born to a nonconsanguineous couple. At 6 months old, he developed a recurrent rash that was punctate, with urticaria or erythematous plaques, which then gradually fused into flakes (**Figures 1B** **, II2**). It was mainly distributed on the limbs, soles, buttocks and face, occasionally accompanied by fever. Generally, the rash lasted for approximately 10 days, while a large area skin rash occurred once every two months. Severe rash flares responded well to cetirizine and corticosteroid treatment. However, the drugs were stopped by the parents due to their concern about the side effects of the drugs. At the age of 4 years, bilateral wrist joint pain occurred without swelling, and the pain was aggravated during activity. The pain could disappear spontaneously after several days without special treatment. Laboratory investigations showed no abnormalities in complete blood cell count, electrolytes, rheumatoid factor, antinuclear antibody, allergen specific IgE, immunoglobulin level, complement components (C3, C4), or liver and renal functions. Regarding inflammatory parameters, C-reactive protein, erythrocyte sedimentation rate and plasma amyloid A were in the normal ranges.

In the family, his father and brother appeared healthy, while his 42- year-old mother (**Figures 1A** **, I1**) had similar symptoms. She reported a long history of periodic arthralgia, occasionally joint swelling after cold exposure. During adolescence, she began to develop urticaria-like rashes on her ankles and thighs after catching colds in the winter. In adulthood, in addition to erythematous papules, right wrist joint and second metatarsophalangeal began to appear joint pain, occasionally accompanied by swelling (**Figures 1B** **, I1**), generally without fever; No specific diagnosis was made. In most cases, the mother has taken nonsteroidal anti-inflammatory drugs (NSAIDs) to relieve the joint pain. The proband and his mother did not develop splenomegaly or bone erosions. Skin biopsy was not performed because they refused.

### Novel NLRC4 Mutation Was Revealed in the Patients

Given the history of recurrent rashes, arthralgia, an autoinflammatory disease was considered. To pursue a molecular diagnosis, the family underwent whole-exome sequencing. Sequencing revealed a heterozygous NLRC4 c.514G>A transition encoding for the p.Gly172Ser variant. The missense mutation was detected in both the proband patient and his mother, but not in healthy members of the family. In addition, this mutation was not found in other databases, including 1000 Genome, ExAC and gnomAD. Both of these results were confirmed by Sanger sequencing (**Figure 1C**). NLRC4 p.Gly172 is highly conserved across many species (**Figure 1D**) and p.Gly172Ser has not previously been documented in the Human Gene Mutation Database(HGMD). The variant was predicted to be likely pathogenic on the basis of conservation, and pathogenicity prediction packages, including Polyphen2, Sorting Intolerant From Tolerant (SIFT), Mutation Taster and PROVEAN.

### The p.G172S Mutation Would Change the Structure of NLRC4

**Figure 2:**
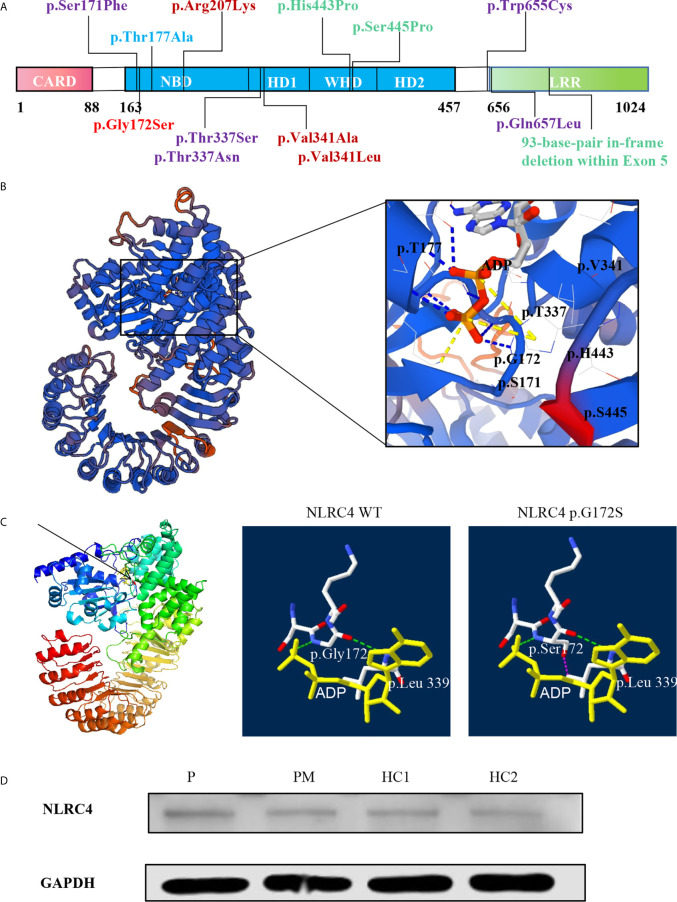
The p.G172S mutation is located in the NBD domain of NLRC4. **(A)** A schematic structure of the NLRC4 protein with the individual domains labeled: CARD, caspase activation and recruitment domain; NBD, nucleotide-binding domain; WHD, winged helix domain; HD1, helicase domain 1; HD2, helicase domain 2; LRR, leucine-rich domain. Mutations with different phenotypes are shown by different colors: NOMID (blue), MAS (purple), AIFEC (dark red), and FACS4 (green). Our patients are shown in red. **(B)** Mapping of Gly172 onto the crystal structure of mouse NLRC4 in the ADP-bound state [Protein Data Bank (PDB) accession 4KXF]. **(C)** The structural impact of mutant Gly172Ser and nearby residues were modeled on the basis of the template of 4KXF from PDB by Swiss-PdbViewer. Hydrogen bonds are shown as green dotted lines. Steric hindrance between residues Ser172 and Leu339 is shown as purple dotted lines. ADP is highlighted with a yellow solid line. **(D)** The protein level of NLRC4 in whole-cell lysate assessed by using western blotting of PBMCs.

NLRC4 consists of an N-terminal caspase activation and recruitment domain (CARD), NBD, HDs; (HD1 and HD2), WHD, and a C-terminal leucine rich repeat domain (LRR) (**Figure 2A**). Based on the crystal structure of the highly homologous mouse NLRC4 protein (16, 17), conformation analysis indicated one hydrogen bond interaction between Gly172 and ADP (**Figure 2B**). In addition, NLRC4 exists in an ADP-dependent autoinhibited monomeric conformation, requiring a stable interaction between its NBD and the WHD mediated by ADP binding, and it was stabilized by the HD1 (19). This mutation substitutes hydrophilic Ser172 for a hydrophobic Gly172 residue, which promotes the formation of the steric hindrance between the Ser172 and Leu339 amino acid residues, represented by the purple dotted line represents (**Figure 2C**). This suggests that this mutation may impair protein function. The p.G172S resides in the NBD domain near to the currently known mutations, which are all located around the ADP-binding regions (**Figure 2B**).

### The p.G172S Mutation Does Not Change the Expression of NLRC4

According to the structural analysis, it was found that this mutation would change the spatial structure of NLRC4. Thus, we next examined whether this mutation would affect the expression of NLRC4. Compared with healthy controls, western blot quantitative analysis showed that the heterozygous mutation in NLRC4 had no significant effect on the expression of the NLCR4 protein in the patients’ PBMCs (**Figure 2D**).

### The p.G172S Mutation Induces an Enhanced Inflammatory Response

**Figure 3:**
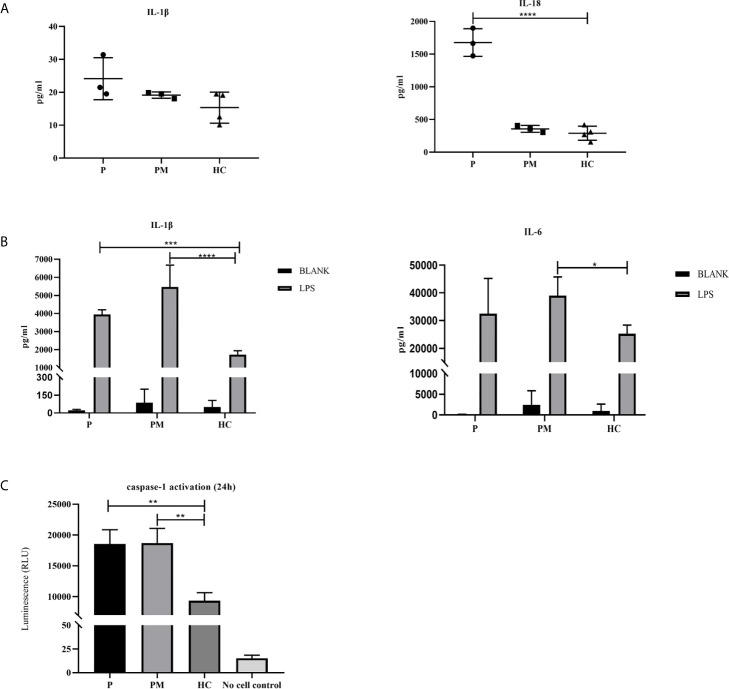
The p.G172S mutation induces a hyperinflammatory response. **(A)** Elevated plasma IL-1β and IL-18 levels were found in the proband patient (P) compared to healthy controls (HCs). **(B)** PBMCs were stimulated with or without 100ng/ml LPS for 24 hours. The secretions of IL-1β and IL-6 were examined in the proband, the mother of proband (PM) and HCs. **(C)** Luminescence assay was performed to quantitate the caspase-1 activation in PBMCs supernatants after LPS stimulation for 24 hours. The level of activated caspase-1 was significantly increased in the patients compared to that in the HCs. The data were pooled from at least 3 independent experiments. *P < .05, **P < .01, ***P < .001, ****P < .0001 by two-tailed t-tests.

Inflammasomes are platforms that integrate danger recognition with the production of the potent proinflammatory cytokines IL-1β and IL-18. The production of cytokines in the plasma was first checked. The level of IL-18 was significantly higher in the proband than in the HCs (**Figure 3A**), but not in his affected mother. We thought this was related to disease activity, because her disease was stable when she was tested. The plasma IL-1β level in the proband was higher than that in the HCs, but there was no significant difference. Then the cytokine responses in the PBMCs upon LPS stimulation were further examined. As expected, the secretion of IL-1β in the two patients was significantly higher than that in healthy controls after LPS stimulation (**Figure 3B**). IL-6 is a downstream molecule of IL-1β (20), and was also checked. Compared with HCs, the secretions of IL-6 in the two patients were higher, with a significant difference demonstrated in the mother of proband (**Figure 3B**). Other cytokines were also tested, including IL-18, TNF-α, MCP, IL-8, IL-10, IL-23, but no statistical difference was shown. Activation of caspase-1 was also measured in the PBMCs culture supernatant using a bioluminescence assay. After 24h of stimulation, the level of activated caspase-1 was significantly increased in the patients compared with the healthy controls (**Figure 3C**). These results suggested that the NLRC4 p.Gly172Ser promote the caspase-1 activation and cytokine production.

### Review of the Literatures About NLRC4 Mutations

To date, twelve NLRC4 mutations have been reported worldwide in the literatures (11–15, 18, 20–28). There were 42 patients from 15 unrelated families, and most of them carried *de novo* mutations. Among them, p.Ser171Phe and p.Thr177Ala were somatic mosaicism mutations, while the others were germline mutations. Except for the 93-base-pair in-frame deletion within exon 5, other NLRC4 mutations were missense heterozygous and most of them are mapped to the critical amino acid residues located at the ADP-bound NBD–WHD–HD1 interaction interface (**Figure 2B**). Four NLRC4 inflammasomopathy phenotypes have been described: AIFEC in 7 cases from 5 families, NLRC4-MAS in 6 cases from 6 families, FCAS4 in 28 cases from 3 families, and NOMID in 1 case from 1 family. All of the information is summarized in **Tables 1**, **2**.

**Table 1:** Summary of previously reported cases with *NLRC4* Mutations.

Variant	Domain	Number of patients	Origin of mutation	Predicted Mutant Type	Age of onset	Country	Phenotype	Biological markers	Treatment	Disease-related mortality	Year	References
p.Gly172Ser	NBD	2 in one family	Inherited	GOF	adolescence, 6 months	China	FCAS4	IL-1β, IL-6, IL-18	NASIDs, corticosteroid, colchicine	Alive at 4 years and 42 years	2019	Present cases
p.Thr337Ser	HD1	One	De novo	GOF	6 months	European	MAS	CRP, IL-1β, MAS IL-18, IL-6, cytotoxic T-cell dysfunction	colchicine, corticosteroids, anakinra	Alive at 7 years	2014	(11)
p.Val341Ala	HD1	3 in one family	De novo, then inherited	GOF	1–2 weeks	USA	AIFEC	CRP, IL-1β, IL-18, IL-2R, MAS, NK cell lymphopenia and disfunction	corticosteroids, cyclosporin	Deceased at 23 days (proband) or alive at 7 and 46 years	2014	(12)
p.His443Pro	WHD	13 in one family	Inherited	GOF	2–3 months	Japanese	FCAS4	IL-17A	without treatment or NSAIDs	Alive into adulthood	2014	(14)
p.Thr337Asn	HD1	One	De novo	GOF	20 days	Caucasian	MAS	CRP, IL-18, INF-γ, MAS, CXCL9, CXCL10	corticosteroids, cyclosporin, anti-IFN-γ monoclonal antibody	Alive at 4.5 months	2015	(21)
p.Ser171Phe	NBD	One	Somatic mosaicism	GOF	In utero	NA	Perinatal MAS and FTV	CRP, IL-2R, MAS	—	Deceased at 2 months	2017	(20)
p.Thr177Ala	NBD	One	Somatic mosaicism	GOF	Birth	Japanese	NOMID	CRP, IL-1β, IL-6, IL-18	colchicine, anakinra	Alive at 19 years	2008 2017	(22, 23)
p.Val341Ala	HD1	One	De novo	GOF	6 weeks	White	AIFEC	CRP, IL-1β, IL-18, IL-6, MAS, cytotoxic T-cell dysfunction	corticosteroids, anakinra, infliximab, cyclosporine, a4b7-integrin inhibition, rhIL-18BP	Alive at 1 year	2017	(13)
p.Ser445Pro	WHD	13 in one family	Inherited	GOF	Infancy to childhood	Dutch	FCAS4	IL-1β, IL-6, IL-10, IL-18, TNF-α, IFN-γ	Anakinra	Alive into adulthood	2017	(15)
P.Arg207Lys	NBD	One	De novo	GOF	8 days	Caucasian	AIFEC	Absent natural killer cell activity, CD25, IL-6, MAS, IL-8 and TNF-α	Corticosteroids, IVIG, Anakinra, BMT	Alive at 6 months	2017	(24)
P.Thr655Cys	LRR	2 unrelated patients	De novo	GOF	11 days, 18 months	NA/China	MAS	CRP, IL-1β, IL-18, IL-2R	corticosteroids, anakinra, IVIG, eculizumab, tocilizumab, IL-18BP	Deceased at age 11 weeks, 18.5 months	2018	(18)
p.Val341Leu	HD1	One	De novo	GOF	12 days	Canada	AIFEC	CRP, IL-1β, IL-18, MAS	corticosteroids, anakinra, rapamycin	Alive at 7 months	2018	(25)
p.Gln657Leu	LRR	One	De novo	GOF	2 weeks	Malaysia	Recurrent fever episodes, skin erythema, and inflammatory arthritis	CRP, IL-18	Colchicine	Alive at 12 years	2019	(26)
p.Val341Ala	HD1	One	De novo	GOF	20 days	Caucasian	AIFEC-associated perirectal abscesses	CRP, PCT, IL-18, MAS	Anakinra	Alive at 2 years	2019	(27)
93-base-pair in-frame deletion within exon 5	LRR	Two	Inherited	GOF	5 to 6 years	NA	FCAS4	NA	Steroids	Alive at 41 years and NA	2020	(28)

HD1, helical domain 1; NBD, nucleotide-binding domain; WHD, winged-helix domain; LRR, leucine-rich repeat domain; GOF, gain-of-function; FCAS, familial cold autoinflammatory syndrome; NOMID, neonatal-onset multisystem inflammatory disease; FTV, fetal thrombotic vasculopathy; AIFEC,autoinflammation with infantile enterocolitis; MAS, macrophage activation syndrome; BMT, bone marrow transplantation; IVIG, intravenous immunoglobulin; NSAIDs, nonsteroidal anti-inflammatory and analgesic drug; PCT, procalcitonin levels; NA, not available.

**Table 2:** The clinical phenotypes decreased order of frequency from the 42 patients published in total with *NLRC4* mutations.

Phenotype	Mutations	Domain of the mutation site	Patients number	Percentage (%)	References
FACS4	p.His443Pro	WHD	13	30.9	(14)
	p.Ser445Pro	WHD	13	30.9	(15)
	93-base-pair in-frame deletion within exon 5	LRR	2	4.7	(28)
AIFEC	p.Val341Ala	HD1	5	11.9	(12, 13, 27)
	p.Val341Leu	HD1	1	2.4	(25)
	p.Arg207Lys	NBD	1	2.4	(24)
NLRC-MAS	p.Thr655Cys	LRR	2	4.8	(18)
	p.Thr337Ser	HD1	1	2.4	(11)
	p.Thr337Asn	HD1	1	2.4	(21)
	p.Ser171Phe	NBD	1	2.4	(20)
	p.Gln657Leu	LRR	1	2.4	(26)
NOMID	p.Thr177Ala	NBD	1	2.4	(22, 23)

FCAS4, familial cold autoinflammatory syndrome; AIFEC, autoinflammation with infantile enterocolitis; NLRC4-MAS, NLRC4-macrophage activation syndrome; NOMID, neonatal-onset multisystem inflammatory disease; NBD, nucleotide-binding domain; WHD, winged helix domain; HD1, helicase domain 1; HD2, helicase domain 2; LRR, leucine-rich domain.

## Discussion

*NLRC4* is located on chromosome 2p22.3, encoding 1024 amino acids. The NLRC4 protein plays important roles in the innate immune response, which forms an inflammasome in response to type 3 secretion system (T3SS) proteins from invading Gram-negative bacteria, such as Salmonella species (29). Mutations in this gene result in autoinflammation disorders with variable clinical manifestations from skin rash to MAS with or without infantile enterocolitis. In this study, we identified a heterozygous mutation resulting in a glycine to serine substitution at the amino acid residue 172, which has not been reported before. Both patients manifested with recurrent urticaria and arthralgia and were treated symptomatically. The amino acid residue Gly172 is highly conserved, and interacts with ADP *via* one hydrogen bond. The mutation promotes the formation of the steric hindrance between Ser172 and Leu339 amino acid residues, suggesting that it could affect NLRC4 function. The levels of plasma IL-1β and IL-18 were higher than in HCs during disease flares in the proband. Functional tests showed that this mutation could enhance the activity of the inflammasome, resulting in an increase of IL-1β and IL-6 secretion and caspase-1 activity after LPS stimulation. However, the levels of IL-18 in P and PM were not significantly higher than that of HCs after LPS stimulation. A possible explanation is that the secretion of IL-18 and IL-1β is not synchronous. As in some CAPS patients with NLRP3 mutations, only the level of IL-1β was found to be elevated, but IL-18 was not (30). In addition, as for NLRC4 mutation, according to the reported literature (15), the levels of IL-18 are different among different patients, even in those with same mutation. Some patients showed increased serum IL-18, but others did not. While the underlying mechanism is not clear so far and need further study.

The literatures on NLRC4-AIDs were systematically reviewed, aiming to characterize the associated clinical features. Of the 42 patients identified so far, the majority were Japanese. The true prevalence of NLRC4-AIDs across ethnicities is poorly understood. Of the 42 patients from 15 unrelated families, 73.8% had a family history, while the remainder had *de novo* mutations. Four clinical phenotypes associated with NLRC4 mutations have been described: AIFEC, NLRC4-MAS, FCAS4 and NOMID.

AIFEC and NLRC4-MAS are two chronic inflammatory disease that are frequently accompanied by life-threatening episodes. All reported AIFEC and NLRC4-MAS patients have required hospitalizations in intensive care units during inflammatory episodes and usually need treatment with a combination of immunosuppressive or biological agents. Four patients died of this disease (12, 18, 20). In severe cases, it is difficult to distinguish between AIFEC/NLRC4-MAS flares and primary hemophagocytic lymphohistiocytosis (HLH). Both manifest with multilineage cytopenias, coagulopathy, hypertriglyceridemia, elevated soluble IL-2 receptor and low cytotoxic cell function (11–13, 20, 21). However, most importantly, the cytotoxic cell function of AIFEC patients returned to normal level after the flare resolved, reflecting an intact granule-dependent cytotoxicity machinery of the cytotoxic cells. Extremely elevated serum IL-18 concentrations are another biomarker in AIFEC/NLRC4-MAS patients that could be used to distinguished them from primary HLH (12, 13, 18, 21, 25–27). Gastrointestinal diseases such as diarrhea, bloody stool and recurrent perianal abscesses also help differentiate AIFEC from HLH (12, 13, 18, 21, 26, 27). Among the 7 AIFEC patients, six of them had a mutation at the acid residue 341, which shows that valine 341 is a hot-spot mutation. In addition, some studies have shown that position 341 is important for closing the lid on the ADP-binding pocket to prevent ADP–ATP exchange (12, 20).

Twenty-eight patients with FACS4 syndrome have been reported in three families, and most of them came from Japanese and Dutch populations (14, 15). Most of them carried mutations in the WHD domain of NLRC4 (p.His443Pro and p.Ser445Pro) (14, 15). These patients showed mild clinical conditions with urticarial rash and arthritis, and even most of them did not require treatment. The symptoms were frequently induced by exposure to cold stimuli. The individuals with the p.Ser445Pro mutation displayed highly elevated plasma IL-18 concentrations (15), similar to our patients, but this was not found in patients with the p.His443Pro mutation (14). As for the p.His443Pro mutation, an *in vitro* study has demonstrated that disruption of the His443-ADP interaction facilitates conformational changes in the WHD and weakens ADP binding, therefore promoting NLRC4 activation (14). In our patients, the p.Gly172Ser mutation promotes the formation of steric hindrance between the Ser172 and Leu339 residues, which would impair protein function, also leading to NLRC4 activation. On the other hand, a functional study suggests that pathologic NLRC4 variants in the WHD may differentially promote caspase 8-mediated cell death (31), which could contribute to the disease development associated with mutations in the WHD domain. For the skin pathological manifestations, skin infiltrates in p.Ser445Pro patient biopsies were lymphohistiocytic, unlike FCAS1 patients whose biopsied skin lesions were characteristically neutrophilic (32).

The NOMID phenotype was only reported in one patient who was NLPR3 mutation-negative but carried a p.Thr177Ala mutation in the NBD domain of NLRC4, which resulted in chronically elevated plasma IL-18. Functional analysis of patient pluripotent stem cell-derived monocytes revealed that the patient had somatic mosaicism of a novel NLRC4 mutation (22, 23).

According to the previously reported cases, there seems to be a genotype and phenotype correlation for NLRC4 mutations. The patients with mutations in the WHD domain presented with mild inflammatory symptoms of FCAS4; whereas severe inflammation, MAS, and AIFEC, developed in those with mutations in the NBD and HD1 domains. However, unlike the reported severe cases with mutations in the NBD domain, we herein identified a mutation in the NBD domain of NLRC4 in two patients, who just presented with recurrent urticaria and arthralgia. This is the first report of FCAS4 associated with a mutation in the NBD domain of NLRC4. Thus, it is still unclear why different mutations of NLRC4 lead to distinct clinical phenotypes, which warrants further investigation.

In summary, we identified a novel mutation in the NBD domain of NLRC4 in two patients with mild clinical phenotype including recurrent urticaria and arthralgia. Our findings demonstrated the diversity of NLRC4 mutations and disease phenotypes, thus expanding the clinical spectrum associated with NLRC4 gain-of-function mutations.

## Data Availability Statement

The datasets presented in this study can be found in online repositories. The names of the repository/repositories and accession number(s) can be found below: https://www.ncbi.nlm.nih.gov/, NM_021209; https://www.uniprot.org/, Q9NPP4; http://www.wwpdb.org/, 4KXF.

## Ethics Statement

The studies involving human participants were reviewed and approved by Institutional Review Board Children’s Hospital Chongqing Medical University. Written informed consent to participate in this study was provided by the participants’ legal guardian/next of kin. Written informed consent was obtained from the individual(s), and minor(s)’ legal guardian/next of kin, for the publication of any potentially identifiable images or data included in this article.

## Author Contributions

HM conceived, designed and guided the study, and revised the manuscript critically. LW, WW, MD, and YL performed the experiments. LW collected and analyzed the data. GS provided the help with the structure analysis. LW wrote the manuscript. XZ and XT checked and revised the manuscript. All authors contributed to the article and approved the submitted version.

## Funding

This work was supported partly by the National Natural Science Foundation of China (Grant number 81971547), the Research Fund for Outstanding Youth Scholar of Chongqing Talents (Grant number CQYC201905003), and the High-level Medical Reserved Personnel Training Project of Chongqing (Grant number 2019181).

## Conflict of Interest

The authors declare that the research was conducted in the absence of any commercial or financial relationships that could be construed as a potential conflict of interest.

## References

[1] Martinon F Aksentijevich I. New Players Driving Inflammation in Monogenic Autoinflammatory Diseases. Nat Rev Rheumatol (2015) 11:11–20. 10.1038/nrrheum.2014.158 25247411

[2] Park H Bourla AB Kastner DL Colbert RA Siegel RM. Lighting the Fires Within: The Cell Biology of Autoinflammatory Diseases. Nat Rev Immunol (2012) 12:570–80. 10.1038/nri3261 PMC4165575 22828911

[3] Hoffman HM Mueller JL Broide DH Wanderer AA Kolodner RD. Mutation of a New Gene Encoding a Putative Pyrin-Like Protein Causes Familial Cold Autoinflammatory Syndrome and Muckle-Wells Syndrome. Nat Genet (2001) 29:301–5. 10.1038/ng756 PMC4322000 11687797

[4] Miceli-Richard C Lesage S Rybojad M Prieur AM Manouvrier-Hanu S Häfner R. CARD15 Mutations in Blau Syndrome. Nat Genet (2001) 29:19–20. 10.1038/ng720 11528384

[5] Jéru I Duquesnoy P Fernandes-Alnemri T Cochet E Yu JW Lackmy-Port-Lis M. Mutations in NALP12 Cause Hereditary Periodic Fever Syndromes. Proc Natl Acad Sci USA (2008) 5:105:1614–9. 10.1073/pnas.0708616105 PMC2234193 18230725

[6] Strowig T Henao-Mejia J Elinav E Flavell R. Inflammasomes in Health and Disease. Nature (2012) 481:278–86. 10.1038/nature10759 22258606

[7] Broz P Dixit VM. Inflammasomes: Mechanism of Assembly, Regulation and Signalling. Nat Rev Immunol (2016) 16:407–20. 10.1038/nri.2016.58 27291964

[8] Feldmann J Prieur AM Quartier P Berquin P Certain S Cortis E. Chronic Infantile Neurological Cutaneous and Articular Syndrome Is Caused by Mutations in CIAS1, a Gene Highly Expressed in Polymorphonuclear Cells and Chondrocytes. Am J Hum Genet (2002) 71:198–203. 10.1086/341357 12032915 PMC384980

[9] Sanchez GA Almeida de Jesus A Goldbach-Mansky R. Monogenic Autoinflammatory Diseases: Disorders of Amplified Danger Sensing and Cytokine Dysregulation. Rheum Dis Clin North Am (2013) 39:701–34. 10.1016/j.rdc.2013.08.001 PMC3888876 24182851

[10] Edwan JH Goldbach-Mansky R Colbert RA. Identification of Interleukin-1β Producing Monocytes That Are Susceptible to Pyronecrotic Cell Death in Patients With Neonatal-Onset Multisystem Inflammatory Disease. Arthritis Rheumatol (2015) 67:3286–97. 10.1002/art.39307 PMC5567735 26245468

[11] Canna SW de Jesus AA Gouni S Brooks SR Marrero B Liu Y. An Activating NLRC4 Inflammasome Mutation Causes Autoinflammation With Recurrent Macrophage Activation Syndrome. Nat Genet (2014) 46:1140–6. 10.1038/ng.3089 PMC4177369 25217959

[12] Romberg N Al Moussawi K Nelson-Williams C Stiegler AL Loring E Choi M. Mutation of NLRC4 Causes a Syndrome of Enterocolitis and Autoinflammation. Nat Genet (2014) 46:1135–9. 10.1038/ng.3066 PMC4177367 25217960

[13] Canna SW Girard C Malle L Jesus AD Romberg N Kelsen J. Life-Threatening NLRC4-Associated Hyper Inflammation Successfully Treated With IL-18 Inhibition. J Allergy Clin Immunol (2017) 139:1698–701. 10.1016/j.jaci.2016.10.022 PMC5846100 27876626

[14] Kitamura A Sasaki Y Abe T Kano H Yasutomo K. An Inherited Mutation in NLRC4 Causes Autoinflammation in Human and Mice. J Exp Med (2014) 211:2385–96. 10.1084/jem.20141091 PMC4235634 25385754

[15] Volker-Touw CML de Koning HD Giltay JC de Kovel CGF van Kempen TS Oberndorff KMEJ. Erythematous Nodes, Urticarial Rash and Arthralgias in a Large Pedigree With NLRC4-Related Autoinflammatory Disease, Expansion of the Phenotype. Br J Dermatol (2017) 176:244–8. 10.1111/bjd.14757 27203668

[16] Hu Z Yan C Liu P Huang Z Ma R Zhang C. Crystal Structure of NLRC4 Reveals Its Autoinhibition Mechanism. Science (2013) 341:172–5. 10.1126/science.1236381 23765277

[17] Hu Z Zhou Q Zhang C Fan SL Cheng W Zhao Y. Structural and Biochemical Basis for Induced Self-Propagation of NLRC4. Science (2015) 350:399–404. 10.1126/science.aac5489 26449475

[18] Moghaddas F Zeng P Zhang Y Schützle H Brenner S Hofmann SR. Autoinflammatory Mutation in NLRC4 Reveals a Leucine-Rich Repeat (LRR)-LRR Oligomerization Interface. J Allergy Clin Immunol (2018) 142:1956–67. 10.1016/j.jaci.2018.04.033 PMC6281029 29778503

[19] Cahill CM Rogers JT. Interleukin (IL) 1beta Induction of IL-6 is Mediated by a Novel Phosphatidylinositol 3-Kinase-Dependent AKT/IkappaB Kinase Alpha Pathway Targeting Activator Protein-1. J Biol Chem (2008) 283(38):25900–12. 10.1074/jbc.M707692200 PMC2533786 18515365

[20] Liang J Alfano DN Squires JE Riley MM Parks WT Kofler J. Novel NLRC4 Mutation Causes a Syndrome of Perinatal Autoinflammation With Hemophagocytic Lymphohistiocytosis, Hepatosplenomegaly. Fetal Thrombotic Vasculopathy, and Congenital Anemia and Ascites. Pediatr Dev Pathol (2017) 20:498–505. 10.1177/1093526616686890 28403691

[21] Bracaglia C. Anti Interferon-Gamma (IFN-γ) Monoclonal Antibody Treatment Ina Patient Carrying an NLRC4 Mutation and Severe Hemophagocytic Lymphohistiocytosis. Pediatr Rheumatol (2015) 13:O68. 10.1186/1546-0096-13-S1-O68

[22] Kawasaki Y Oda H Ito J Niwa A Tanaka T Hijikata A. Identification of a High-Frequency Somatic NLRC4 Mutation as a Cause of Autoinflammation by Pluripotent Cell-Based Phenotype Dissection. Arthritis Rheumatol (2017) 69:447–59. 10.1002/art.39960 27788288

[23] Kashiwagi Y Kawashima H Nishimata S Nagao R Watanabe K Takekuma K. Extreme Effificiency of Anti-Interleukin-1 Agent (Anakinra) in a Japanese Case of CINCA Syndrome. Clin Rheumatol (2008) 27:277–9. 10.1007/s10067-007-0734-7 17891446

[24] Goddard AVG. A Novel NLRC4 Mutation Treated With Bone Marrow Transplantation. Seattle: Clinical Immunology Society (2017). Available at: https://cis.confex.com/cis/2017/webprogram/Paper5388.html.

[25] Barsalou J Blincoe A Fernandez I Dal-Soglio D Marchitto L Selleri S. Rapamycin as an Adjunctive Therapy for NLRC4 Associated Macrophage Activation Syndrome. Front Immunol (2018) 9:2162. 10.3389/fimmu.2018.02162 30319625 PMC6166634

[26] Chear CT Nallusamy R Canna SW Chan KC Baharin MF Hishamshah M. A Novel *De Novo* NLRC4 Mutation Reinforces the Likely Pathogenicity of Specifific LRR Domain Mutation. Clin Immunol (2019) 211:108328. 10.1016/j.clim.2019.108328 31870725

[27] Siahanidou T Nikaina E Kontogiorgou C Tzanoudaki M Stefanaki K Skiathitou AV. Autoinflammation With Infantile Enterocolitis Associated With Recurrent Perianal Abscesses. J Clin Immunol (2019) 39:237–40. 10.1007/s10875-019-00611-w 30864118

[28] Jeskey J Parida A Graven K Hostoffer R. Novel Gene Deletion in NLRC4 Expanding the Familial Cold Inflammatory Syndrome Phenotype. Allergy Rhinol (Providence) (2020) 11:2152656720928062. 10.1177/2152656720928062 32537258 PMC7268108

[29] Duncan JA Canna SW. The NLRC4 Inflammasome. Immunol Rev (2018) 281:115–23. 10.1111/imr.12607 PMC5897049 29247997

[30] Kubota K Ohnishi H Teramoto T Matsui E Murase K Kanoh H. In Vitro Analysis of the Functional Effects of an NLRP3 G809S Variant With the Co-Existence of MEFV Haplotype Variants in Atypical Autoinflammatory Syndrome. J Clin Immunol (2013) 33(2):325–34. 10.1007/s10875-012-9805-x 23015306

[31] Raghawan AK Sripada A Gopinath G Pushpanjali P Kumar Y Radha V. A Disease-Associated Mutant of NLRC4 Shows Enhanced Interaction With SUG1 Leading to Constitutive FADD Dependent Caspase-8 Activation and Cell Death. J Biol Chem (2017) 292:1218–30. 10.1074/jbc.M116.763979 PMC5270468 27974463

[32] Kolivras A Theunis A Ferster A Lipsker D Sass U Dussart A. Cryopyrin-Associated Periodic Syndrome: An Autoinflammatory Disease Manifested as Neutrophilic Urticarial Dermatosis With Additional Perieccrine Involvement. J Cutan Pathol (2011) 38:202–8. 10.1111/j.1600-0560.2010.01638.x 21062341

